# Post-COVID-19 related bleeding at unusual sites: add paravertebral hematomas to the list

**DOI:** 10.11604/pamj.2023.45.122.40947

**Published:** 2023-07-13

**Authors:** Nikolaos Sabanis, Achilleas Betsikos

**Affiliations:** 1 Department of Nephrology, General Hospital of Trikala, Trikala, Greece,; 2 Department of Internal Medicine, General Hospital of Trikala, Trikala, Greece

**Keywords:** Paravertebral hematomas, post-COVID-19 bleedings, soft tissue hematoma

## Image in medicine

A 76-year-old man with a history of end-stage renal disease on hemodialysis and heparin-induced thrombocytopenia was affected by moderate COVID-19 pneumonia. He was supported with low-flow nasal cannula oxygen and received therapy with dexamethasone, ceftriaxone, remdesivir, clopidogrel and fondaparinux at prophylactic dose. During hospitalization, he experienced a new-onset of atrial fibrillation and the initial prophylactic dose of fondaparinux switched to therapeutic one. Thereafter, he remained uneventful and discharged home. After 14 days, the patient complained of progressively worsening back pain accompanied by clinical signs of hemorrhagic shock and an extended ecchymosis on the right paravertebral region. Laboratory tests revealed severe anemia with no evidence of thrombocytopenia, hypofibrinogenemia, disseminated intravascular coagulopathy or prolonged anti-Xa activity. Chest and abdominopelvic computed tomography showed multiple bilateral hematomas located in the erector spinae muscles, with no evidence of active bleeding. Conservative treatment was efficient and gradual absorption of hematomas was observed. Non-traumatic soft tissue hematomas at unusual sites have been described in limited cases of COVID-19 affected patients with multiple underlying comorbidities, early in the course of the disease and predominantly in association with critical illness and systemic anticoagulation use. Therefore, this is a unique case of delayed onset of multiple paravertebral hematomas in association with treatment with fondaparinux, in a post-COVID-19 hemodialysis patient.

**Figure 1:**
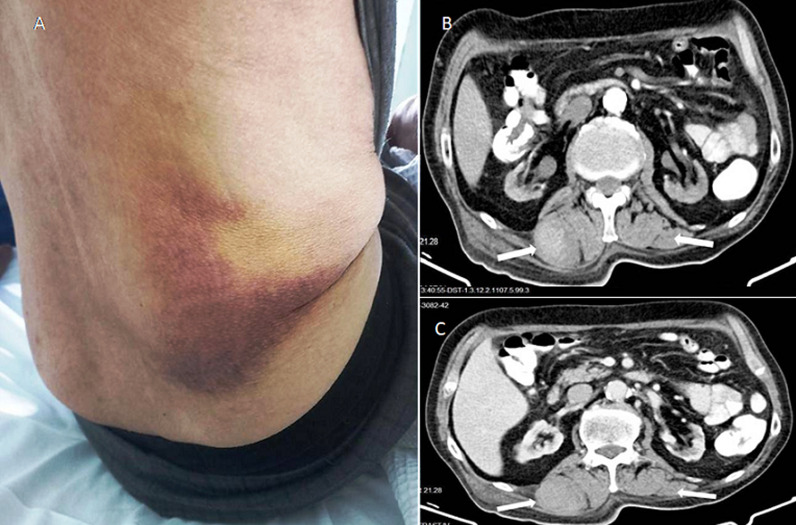
A) extended ecchymosis on the right flank region; B, C) multiple bilateral hematomas in the erector spinae muscles

